# Early *H*ydrogen–*O*xygen Gas Mixture Inhalation in Patients with *A*neurysmal Subarachnoid Hemorrhage (HOMA): study protocol for a randomized controlled trial

**DOI:** 10.1186/s13063-024-08231-5

**Published:** 2024-06-11

**Authors:** Fa Lin, Runting Li, Yu Chen, Jun Yang, Ke Wang, Yitong Jia, Heze Han, Qiang Hao, Guangzhi Shi, Shuo Wang, Yuanli Zhao, Xiaolin Chen

**Affiliations:** 1 Department of Neurosurgery, Beijing Tiantan Hospital, Capital Medical University, No. 119 South Fourth Ring West Road, Fengtai District, Beijing, 100070 China; 2 China National Clinical Research Center for Neurological Diseases, Beijing, China; 3 Department of Critical Care Medicine, Beijing Tiantan Hospital, Capital Medical University, Beijing, China; 4 Stroke Center, Beijing Institute for Brain Disorders, Beijing, China; 5 Beijing Key Laboratory of Translational Medicine for Cerebrovascular Disease, Beijing, China

**Keywords:** Aneurysmal subarachnoid hemorrhage, Randomized controlled trial, Hydrogen–oxygen gas, Delayed cerebral ischemia, Cerebral vasospasm

## Abstract

**Background:**

Aneurysmal subarachnoid hemorrhage (aSAH) is a life-threatening neurosurgical emergency with a high mortality rate. Delayed cerebral ischemia (DCI) and cerebral vasospasm (CVS) are delayed products of early brain injury (EBI), which may constitute the principal determinant of an unfavorable patient prognosis. Consequently, the mitigation of DCI and CVS assumes paramount significance in the pursuit of enhanced patient outcomes. However, except for oral nimodipine, there is no effective therapy available in the current guideline. Hence, the exigency arises to proffer novel treatment paradigms. The diversity of hydrogen therapeutic targets has been largely reported in basic research, unveiling its latent capacity to ameliorate EBI in aSAH patients.

**Methods:**

Early *H*ydrogen–*O*xygen Gas *M*ixture *I*nhalation in Patients with Aneurysmal Subarachnoid Hemorrhage (HOMA), a single-center, prospective, open-labeled, randomized controlled clinical trial, endeavors to evaluate the efficacy and safety of hydrogen–oxygen gas mixture inhalation therapy in aSAH patients. A cohort of 206 patients will be randomized to either hydrogen–oxygen gas mixture inhalation group (8 h per day, 3 L/min, hydrogen concentration of 67%, oxygen concentration of 33%) or oxygen inhalation group (8 h per day, 3 L/min, oxygen concentration of 33%) within 72 h after aSAH and treated for 7 days in the ICU ward. The primary outcomes are the incidence of DCI and CVS during hospitalization.

**Discussion:**

The HOMA aims to evaluate the effectiveness of hydrogen–oxygen gas mixture inhalation therapy in preventing DCI or CVS and improving outcomes in aSAH patients. Notably, this is the first large-scale trial of hydrogen therapy in aSAH patients. Given that the Chinese population represents a significant portion of the global population and the increasing incidence of stroke due to aging, optimizing patient care is vital. Given the current challenges in aSAH patient outcomes, initiating more prospective clinical trials is essential. Recent research has shown hydrogen’s therapeutic potential, aligning with EBI in aSAH, driving our exploration of hydrogen therapy’s mechanisms in post-aneurysm rupture damage.

**Ethics and dissemination:**

The protocol for the HOMA study was approved by the Ethics Committee of Beijing Tiantan Hospital, Capital Medical University (KY 2022–020-02). All results of the present study will be published in peer-reviewed journals and presented at relevant conferences.

**Trial registration:**

ClinicalTrials.gov NCT05282836. Registered on March 16, 2022.

**Supplementary Information:**

The online version contains supplementary material available at 10.1186/s13063-024-08231-5.

## Strengths and limitations of this study

- This study endeavors to amalgamate hydrogen–oxygen gas inhalation with the concept of early brain injury subsequent to aneurysmal subarachnoid hemorrhage (aSAH), elucidating the prospective mechanisms underlying the efficacy of hydrogen–oxygen gas inhalation in the treatment of aSAH.
- Employing a legal hydrogen–oxygen generator equipped with a nebulizer for aSAH patients, this study not only addresses the safety concerns related to hydrogen preparation but also assures the secure clinical application of hydrogen therapy.
- The study’s primary objective is to explore the impact of hydrogen–oxygen gas inhalation therapy on the mitigation of delayed cerebral ischemia (DCI) and cerebral vasospasm (CVS), with a secondary aim of ascertaining whether this therapy can ameliorate the long-term prognosis of individuals afflicted by aSAH.
- It is imperative to note that this investigation is a single-center study. Consequently, should affirmative conclusions emerge, external validation will be imperative to bolster the reliability and generalizability of the findings.

## Background

Aneurysmal subarachnoid hemorrhage (aSAH) represents a prevalent neurosurgical emergency, characterized by a mortality rate spanning from 22 to 50%. Even among patients receiving optimal medical care, 40% of survivors contend with enduring physical disability or cognitive impairment, imposing a substantial healthcare burden on both families and the national medical infrastructure [1]. The concept of early brain injury (EBI), elucidating the pathophysiological changes unfolding within the brain during the initial 72-h post-aneurysm rupture, is now widely regarded as the primary driver of adverse patient outcomes [2, 3]. At this stage, marked by hemorrhagic release and accumulation, acute reactive hyperemia, and transient cerebral ischemia, the brain undergoes a cascade of responses, encompassing energy depletion, ionic perturbations, nitric oxide depletion, heightened endothelin-1 levels, inflammation, and oxidative stress [4–7]. Certain among these factors have been linked to grave complications, including delayed cerebral ischemia (DCI) and cerebral vasospasm (CVS), thereby elevating the peril of an unfavorable patient prognosis.[8].

While clinicians prioritize the prevention and management of DCI and CVS, it is imperative not to overlook other potential contributors to adverse patient outcomes. A recent review delved into the association between treating DCI or CVS and improving outcomes, with disheartening findings [9]. The occurrence of DCI and CVS may only encapsulate a fraction of the multifaceted mechanisms underpinning EBI-induced injury. Relying solely on the prevention and treatment of DCI or CVS presents two dilemmas: firstly, it may fall short in averting or reversing DCI or CVS, and secondly, even if DCI or CVS can be successfully managed, there may be no means to fully counterbalance the impact of other EBI mechanisms on patient prognosis. Thus, our hypothesis posits that an all-encompassing approach, targeting the spectrum of therapeutic objectives offered by EBI, is of paramount importance in averting severe complications and ameliorating patient outcomes.

Hydrogen, the most diminutive and copious element in our realm, possesses the attributes of rapid diffusion, non-toxicity, and benignity. In 2007, Ohsawa et al. pioneeringly postulated that inhaling a 2% hydrogen blend could selectively quell hydroxyl radicals and peroxynitrites, thereby significantly ameliorating the oxidative stress response induced by cerebral ischemia/reperfusion in a rat model of such ischemia–reperfusion [10]. Since that seminal discovery, hydrogen therapy has surged into the limelight, emerging as a focal point in disease treatment research [11–13]. As substantiated by prior investigations, hydrogen demonstrates its potency in mitigating oxidative stress-induced inflammatory responses, as evidenced by its capacity to down-regulate IL-1β, IL-6, chemokines, and TNF-α in various injury models [14–16]. Furthermore, hydrogen assumes roles in anti-apoptosis, anti-allergy, autophagy regulation, and enhanced energy metabolism [17, 18]. Beyond these chemical effects, its minuscule molecular weight enables unhindered penetration of brain cells via the blood–brain barrier, permeation of biofilms, and action upon therapeutic targets such as mitochondria. Considering these therapeutic facets, hydrogen manifests itself as ideally suited to combat the evolution of EBI following aSAH, offering distinctive advantages in cerebral protection subsequent to this condition. Preclinical evidence has demonstrated the efficacy of hydrogen and hydrogen–oxygen gas mixtures in improving behavioral outcomes and prognosis in murine models of experimental subarachnoid hemorrhage [19–22].

Nonetheless, the widespread clinical application of hydrogen has been hindered by challenges associated with its production and storage. Fortunately, China has recently achieved independent intellectual property rights and authorized hydrogen inhalation equipment, capable of generating a hydrogen–oxygen mixture via water electrolysis. Consequently, the time is ripe for an exploration of hydrogen’s role in aSAH treatment.

## Methods/design

**Fig. 1:**
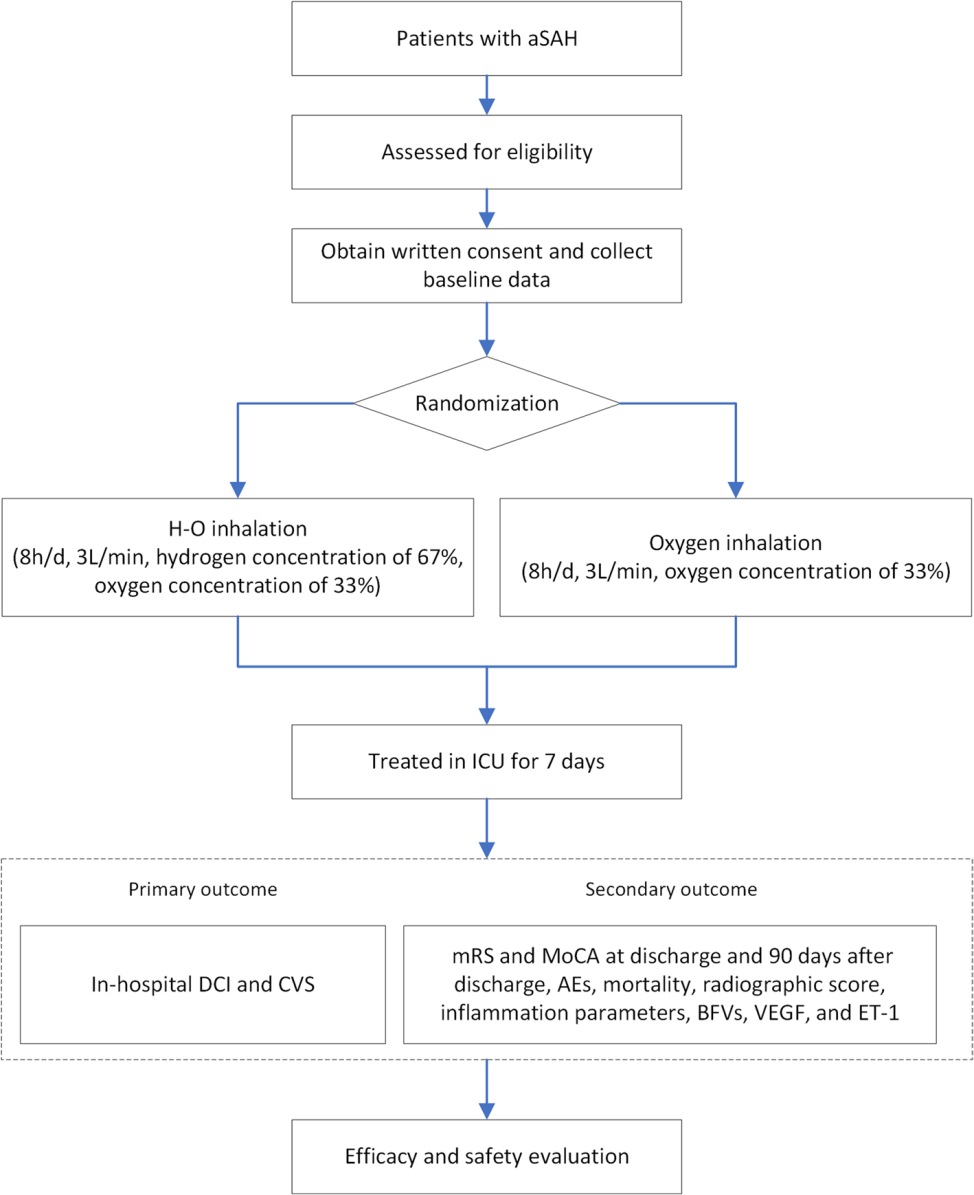
Flow diagram of the study

Early *H*ydrogen–*O*xygen Gas *M*ixture Inhalation in Patients with *A*neurysmal Subarachnoid Hemorrhage: a randomized controlled trial (HOMA Trial) is a single-center, prospective, open-labeled, randomized controlled clinical trial in patients with acute aSAH at Beijing Tiantan Hospital. All patients are managed in alignment with the guidelines (such as maintaining euvolemia, avoiding hypo- and hypervolemia and repletion of volume and sodium losses, systolic blood pressure < 160 mmHg before aneurysm securement, and premorbidity blood pressure after aneurysm securement) [23]. Figure 1 shows the flow diagram of the study.

### Patient population

HOMA is set to recruit 206 participants, evenly divided with 103 allocated to each treatment arm from China, to successfully execute the trial.

### Inclusion criteria

- Age ≥ 18 years old;
- Emergency admission;
- Less than 48 h from the rupture to the admission and less than 24 h from the admission to surgical clipping or endovascular treatment;
- Written consent to participate in the study.

### Exclusion criteria

- Previous SAH;
- History of neurosurgery due to any reasons;
- Physical disability or cognitive impairment due to any previous disease;
- Treatments including external ventricular drainage, lumbar puncture, angiography, intubation, and/or mechanical ventilation at other hospitals before presentation to our hospital;
- Pregnancy or breastfeeding;
- Any condition that imposes hazards to the patient due to treatment;
- Patients with foreseeable difficulties in attending follow-ups adequately;
- Patients with oxygen saturation < 88%;
- If oxygen saturation (SpO2) < 88% for patients in both groups, additional oxygen supplementation will be titrated to maintain SpO2 ≥ 88% by another oxygen concentrator. Patients will be allowed to withdraw from the study if SpO2 is still less than 88% when the total oxygen flow rate reached 7.0 L/ min.

### Randomization

Patients will undergo randomization performed by an intensive critical care (ICU) physician. Allocation will follow a 1:1 ratio through a stratified randomization approach. Allocation will be determined utilizing a minimization method of dynamic allocation, taking into consideration the preprocedural World Federation of Neurological Societies (WFNS) grade as a decisive factor.

### Treatment

Both hydrogen–oxygen mixture and oxygen are introduced via a nasal catheter.

- Experimental arm: hydrogen–oxygen gas mixture inhalation therapy (8 h/day, 3 L/min, hydrogen concentration of 67%, oxygen concentration of 33%) daily for 1–7 days in the ICU ward.
- Control arm: oxygen gas inhalation therapy (8 h/day, 3 L/min, oxygen concentration of 33%) daily for 1–7 days in the ICU ward.d

The postoperative intervention of this study is administered by the assigned on-duty neurosurgeons in adherence to standardized operating protocols to enhance compliance.

### Primary outcomes

- In-hospital DCI. DCI is diagnosed based on the following criteria: the emergence of new focal neurological deficits or a global neurological decline (defined as cerebral infarction identified on CT or MRI, after exclusion of procedure-related infarctions, and a decrease of ≥ 2 points on the Glasgow Coma Scale (GCS)) persisting for over 2 h [24]. This diagnosis is made after ruling out intracranial hemorrhage, hydrocephalus, seizures, metabolic imbalances, and infections, with or without radiographic evidence of cerebral vasospasm.
- In-hospital CVS. The diagnostic criteria for CVS encompass transcranial Doppler (TCD) sonography measurements (mean flow velocity exceeding 150 cm/s and/or Lindegaard index surpassing 3, and/or a TCD velocity increase exceeding 50 cm/s within 24 h), or cranial computed tomography (CT) angiography revealing vessel constriction exceeding 66%.

### Secondary outcomes and exploratory endpoints

- The functional outcome will be evaluated by the modified Rankin scale (mRS) at discharge and 3 months after discharge via outpatient appointment.
- The cognitive outcome will be evaluated using the Montreal Cognitive Assessment (MoCA) at discharge and 3 months after discharge via outpatient appointment.
- Adverse events (AEs), including in-hospital pneumonia, intracranial infection, deep vein thrombosis (DVT), and stress ulcer bleeding.
- Mortality at discharge and 3 months after discharge.
- Radiographic score, including subarachnoid hemorrhage early brain edema (SEBES) score, modified Fisher scale (mFS) grade, Graeb score.
- Inflammation parameters, including white blood cell (WBC), neutrophil (NEUT), C-reactive protein (CRP), procalcitonin (PCT), IL-2, IL-6, IL-8, IL-10, TNF-α.
- Average blood flow velocities (BFVs) of anterior, posterior, and middle cerebral arteries.
- Levels of vascular endothelial growth factor (VEGF) and endothelin-1 (ET-1).

The detailed schedule of study assessments is shown in Table 1.

**Table 1:** Schedule of enrollment, interventions, and assessments

Variables	Visit 1	Visit 2	Visit 3	Visit 4	Visit 5	Visit 6	Visit 7	Visit 8	Visit 8	Visit 9
	Admission within 72 h	Day 1 in ICU	Day 2 in ICU	Day 3 in ICU	Day 4 in ICU	Day 5 in ICU	Day 6 in ICU	Day 7 in ICU	Discharge	90 days
Participants										
Eligibility screen	×									
Informed consent	×									
Randomization		×								
Demographic data	×									
Interventions										
Surgical clipping or endovascular treatment	×									
Inhalation		×								
Assessments										
Modified Rankin scale	×								×	×
GCS	×	×	×	×	×	×	×	×	×	×
WFNS	×									
Hunt and Hess	×									
Modified Fisher	×									
Vital signs	×	×	×	×	×	×	×	×		
Inflammation parameters	×	×		×		×		×	×	×
VEGF	×	×		×		×		×	×	×
ET-1	×	×		×		×		×	×	×
Head CT	×	×						×	×	×
Head CTA + CTP	×								×	×
TCD				×			×			
MoCA									×	×
Adverse events		×	×	×	×	×	×	×	×	×
Detailed ICU therapy								×		
Inhalation time		×	×	×	×	×	×	×		
Inhalation volume		×	×	×	×	×	×	×		

*GCS* Glasgow Coma Scale, *WFNS* World Federation of Neurological Societies, *VEGF* vascular endothelial growth factor, *ET-1* endothelin-1, *TCD* transcranial Doppler

### Data collection and monitoring

The two clinicians will undergo standardized training for data collection. Their proficiency will be assessed prior to commencing data collection, and the inter-rater reliability between the two clinicians will be evaluated. Study data will be gathered using an electronic clinical report form in Castor EDC, a data management system compliant with good clinical practices. The Data Safety Monitoring Board (DSMB), comprising two independent clinicians and one methodologist, will scrutinize all AEs reported by enrolled patients from the time of consent up to 12 months after discharge. The DSMB will oversee the overall conduct of the trial and possesses the authority to modify or halt the study based on a safety data review. DSMB meetings will be convened annually via online video conferencing until the study’s completion. The Trial Steering Group is responsible for running the trial day-to-day and providing organizational support, consisting of a principal investigator, research assistant, recruitment, and assignment personnel. They will meet to review every 3 months and the ethics committee will perform an annual follow-up review.

### Protocol amendment

The principal investigator will be accountable for any protocol amendments and render the final determination. Should any critical protocol modifications arise (e.g., changes to eligibility criteria, outcomes, analyses), the principal investigator will communicate and secure approval from the local Medical Ethics Committee before implementation. Any revised protocol will be updated on the ClinicalTrials.gov registry website.

### Safety considerations

**Fig. 2:**
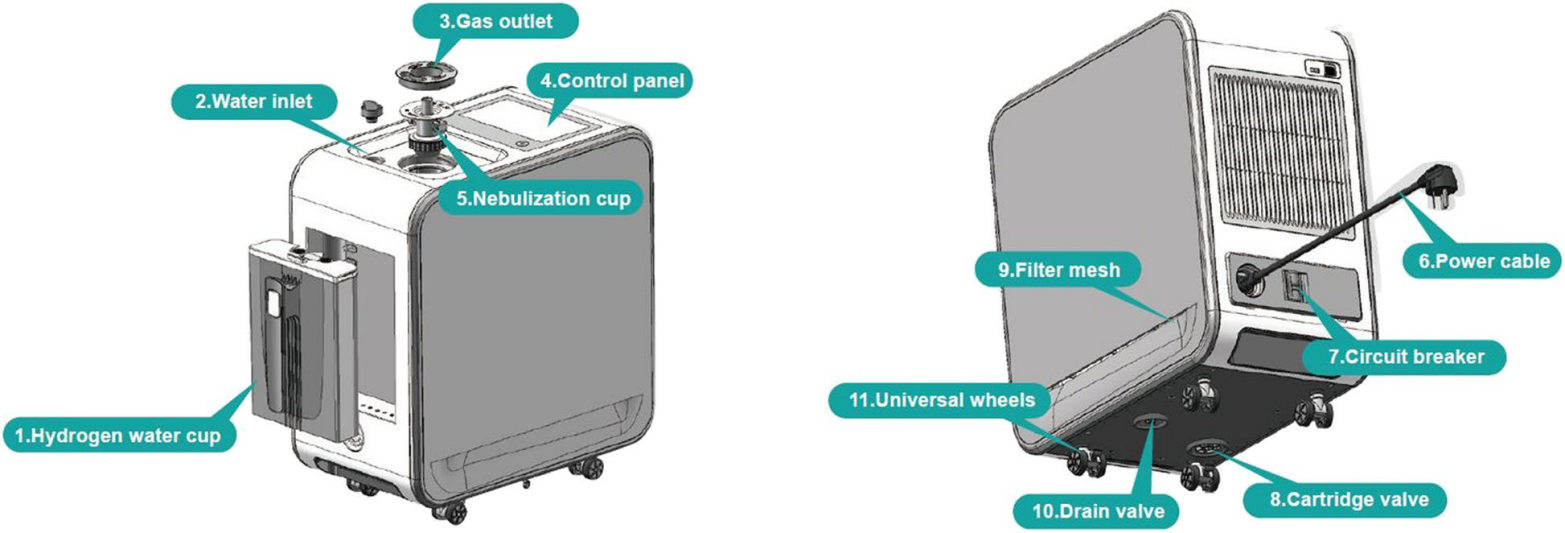
A schematic diagram of the hydrogen/oxygen generator (model AMS-H-03). Courtesy of Shanghai Asclepius Meditec Co., Ltd. (Shanghai, China)

The apparatus employed in this study is hydrogen–oxygen generator with nebulizer (AMS-H-03, Shanghai Asclepius Meditech Co., Ltd. China) (Fig. 2). A comprehensive review has furnished in-depth insights into the device’s underlying principles, safety features, and its method of application within the trial [25]. In addition to the device’s specialized design, stringent safety measures will be implemented, including the conduction of the study within a well-ventilated environment and the utilization of an ambient hydrogen concentration monitor to ensure that the hydrogen concentration in the surroundings remains below 4%.

### Sample size estimates

PASS (Version 15.0) software was utilized for sample size determination. The primary study outcomes encompass the incidence of in-hospital DCI and CVS, both exhibiting nearly equivalent prevalence rates. Drawing from our current retrospective and prospective data, we anticipate a prevalence of 23% in the experimental group and 45% in the control group. With a significance level (*α*) of 0.05 and a desired statistical power of 90%, the necessary sample size per study arm to discern this difference is calculated to be 103 patients.

### Statistical analysis

We will conduct data analysis in accordance with the intention-to-treat principle. Continuous variables will be presented as mean ± standard deviation (SD) or median along with interquartile range (IQR). Categorical variables were summarized as frequency (percentage). Continuous variables will be assessed using either the independent Student’s *t*-test (assuming normal distribution) or the Mann–Whitney *U* test. Dichotomized variables will be compared utilizing the Pearson chi-square test or Fisher’s exact test. To mitigate preoperative confounding factors, a multiple regression analysis will be employed to compare outcomes between the two groups. The results will be reported as adjusted odds ratios (OR) accompanied by 95% confidence intervals (CI), with OR values below 1 indicating a treatment effect in favor of hydrogen–oxygen mixture therapy. Postoperative follow-up assessments at designated time intervals are imperative, and the absence of data should not be anticipated. Statistical significance will be defined as a threshold of *P* < 0.05.

Subgroups analysis will be performed, including subgroup by age, sex, WFNS, mFS, and treatment modality. Subgroup analyses are limited to the primary efficacy endpoint and safety endpoint only. Separate logistic models will be applied to each subgroup during subgroup analysis.

There will be no interim analyses in this trial.

### Patient and public involvement

Patients or the public have not participated in the design or execution of our research. Upon study completion, a manuscript will be prepared to present the trial findings. The final study results will be disseminated to all participants through the methods communicated at enrollment (mobile phone, WeChat, email, etc.).

### Study timeline

We anticipate that the study will span a duration of 24 months. Commencing in mid-June 2022, patient enrollment is expected to span a period of 18 months. An additional 6 months will be allocated for the meticulous tasks of data cleaning, in-depth analysis, and the preparation of the manuscript.

## Discussion

HOMA represents a single-center, prospective, open-label, and randomized controlled clinical trial, aimed at assessing the potential of hydrogen–oxygen gas mixture inhalation therapy in averting DCI or CVS while ameliorating outcomes in patients afflicted by aSAH. It is worth noting that this constitutes the first large-scale clinical trial of hydrogen therapy as applied to aSAH patients.

The Chinese population accounts for approximately one-fifth of the global population. With the advent of the aging demographic epoch, the incidence of stroke has been on a steady rise, posing a significant menace to public health. Consequently, the optimization of patient care has assumed paramount importance within the healthcare system.

Given the current impasse in substantially improving outcomes for aSAH patients, clinicians should be prompted to initiate additional prospective clinical trials. Recent years have witnessed the unequivocal validation of hydrogen has therapeutic potential in basic research [26, 27]. We posit that hydrogen’s therapeutic attributes closely align with the concept of EBI following aSAH. Therefore, we embarked on the utilization of hydrogen therapy in aSAH patients, with the aim of elucidating potential mechanisms underlying post-aneurysm rupture bodily damage.

## Trial status

This research protocol version 1.0 (2022/05/07) is approved, and recruitment of patients for this HOMA trial has begun in September of 2022 and is expected to complete in March of 2024.

### Supplementary Information

Supplementary Material 1.

## Abbreviations

aSAH: Aneurysmal subarachnoid hemorrhage
DCI: Delayed cerebral ischemia
CVS: Cerebral vasospasm
EBI: Early brain injury
OR: Odds ratios
CI: Confidence intervals
DSMB: Data Safety Monitoring Board
GCS: Glasgow Coma Scale
WFNS: World Federation of Neurological Societies
VEGF: Vascular endothelial growth factor
ET-1: Endothelin-1
TCD: Transcranial Doppler
SEBES: Subarachnoid Hemorrhage Early Brain Edema
mFS: Modified Fisher scale
CRP: C-reactive protein
